# To investigate the correlation between normal fetal biventricular myocardial function and gestational age using velocity vector imaging

**DOI:** 10.3389/fcvm.2023.920965

**Published:** 2023-03-23

**Authors:** Min Hou, Xiao-Jing Duan, Yan An, Ji-Peng You, Liang-Liang Bi, Xuan Zhou, Jie Wan, Yi Qu

**Affiliations:** ^1^ Department of Ultrasonography, Affiliated Hospital of Hebei University, Baoding, China; ^2^ Department of Obstetrics, Affiliated Hospital of Hebei University, Baoding, China; ^3^ Department of Emergency Medicine, Affiliated Hospital of Hebei University, Baoding, China; ^4^ Department of Nursing, Children’s Hospital of Hebei Province, Shijiazhuang, China

**Keywords:** ultrasonic velocity vector imaging, normal fetus, biventricular strain, strain rate, myocardial deformability

## Abstract

**Objective:**

The aim of this study was to evaluate the left and right ventricular segmental and global myocardial function of normal fetuses using velocity vector imaging and explore the correlation between global myocardial function parameters and gestational age.

**Methods:**

A total of 127 normal fetuses were selected and divided into five groups according to gestational age for the measurement of their left and right ventricular segmental and global velocity, strain, and strain rate. This study also explored the change trend in the global myocardial function parameters at different gestational ages and analyzed its correlation with gestational age.

**Results:**

The peak velocities of the biventricular segments of the normal fetuses showed a decreasing trend from the basal to the middle to the apex segment, and the differences were statistically significant (*P* < 0.05). However, the strain and peak strain rate between adjacent segments showed no significant differences (*P* > 0.05). The peak global velocity of both ventricles increased with the gestational age, and it was moderately correlated with gestational age; however, the correlation of strain and peak strain rate with gestational age was not statistically significant (*P* > 0.05).

**Conclusion:**

In normal fetuses, the peak myocardial velocity of the biventricular segments showed a decreasing trend from the basal to the apical segment. The global peak myocardial velocity was linearly correlated with gestational age; however, the global strain and peak strain rate did not change as gestational age increased, indicating that the myocardial deformability of the fetus' ventricles was constant in the middle and late trimesters.

## 1. Introduction

Ventricular function is an important prognostic factor of cardiopulmonary pathology, but it is usually difficult to evaluate the left and right ventricular function of a fetus. As technology developed, echocardiography could quantitatively evaluate the global cardiac function and quantitatively analyze and evaluate the motion of the segmental ventricular wall. Velocity Vector Imaging (VVI) (1–4) is a newer method that is utilized to measure the cardiac velocity and deformation independent of Doppler and without direction constraint, and it can automatically determine the center of motion without considering the heartbeat, showing a high detection repeatability (5). In this study, normal fetuses are measured for the segmental and global myocardial function of their left and right ventricles using VVI. An analysis of the correlation between the parameters and gestational age is performed, and the differences between the parameters of the left and right ventricles are compared. In addition, the VVI parameter values, used in assessing fetal myocardial function, are evaluated.

## 2. Material and methods

### 2.1. Subjects

A total of 127 pregnant women, visiting the Affiliated Hospital of Hebei University from January 2016 to April 2018, were selected as the subjects. The fetuses were divided into five groups according to the gestational age: Group 1: 16–19^+6^ weeks; Group 2: 20–23^+6^ weeks; Group 3: 24–27^+6^ weeks; Group 4: 28–31^+6^ weeks; and Group 5: 32–35^+6^ weeks. Inclusion criteria: Pregnant women who had no family history of congenital heart disease or systemic diseases such as high blood pressure, diabetes, and phenylketonuria, no teratogenic exposure history, no history of taking chemicals (such as alcohol, anticonvulsants, lithium carbonate, vitamin A, and warfarin), no infectious diseases (rubella virus, cytomegalovirus, coxsackie virus, and parvovirus), and no history of irradiation. All pregnant women had normal menstrual cycles before pregnancy, or the gestational age was consistent with the gestational weeks confirmed by ultrasound during early pregnancy. All fetuses were single pregnancies, without cardiac structural malformations and arrhythmias. Exclusion criteria: Pregnant women suffer from congenital heart disease, hypertension, diabetes and other systemic diseases, taking drugs and suffering from infectious diseases. Multiple births. Fetal heart structural abnormality or arrhythmia.

### 2.2. Instruments used

A GE Voluson E8/E10 color Doppler diagnostic apparatus, with a probe frequency of 2–5 MHz. Two-dimensional (2D) strain analysis software (for the VVI images; TomTec) was used for offline post-processing analysis.

### 2.3. Methods and testing indexes

**Figure 1:**
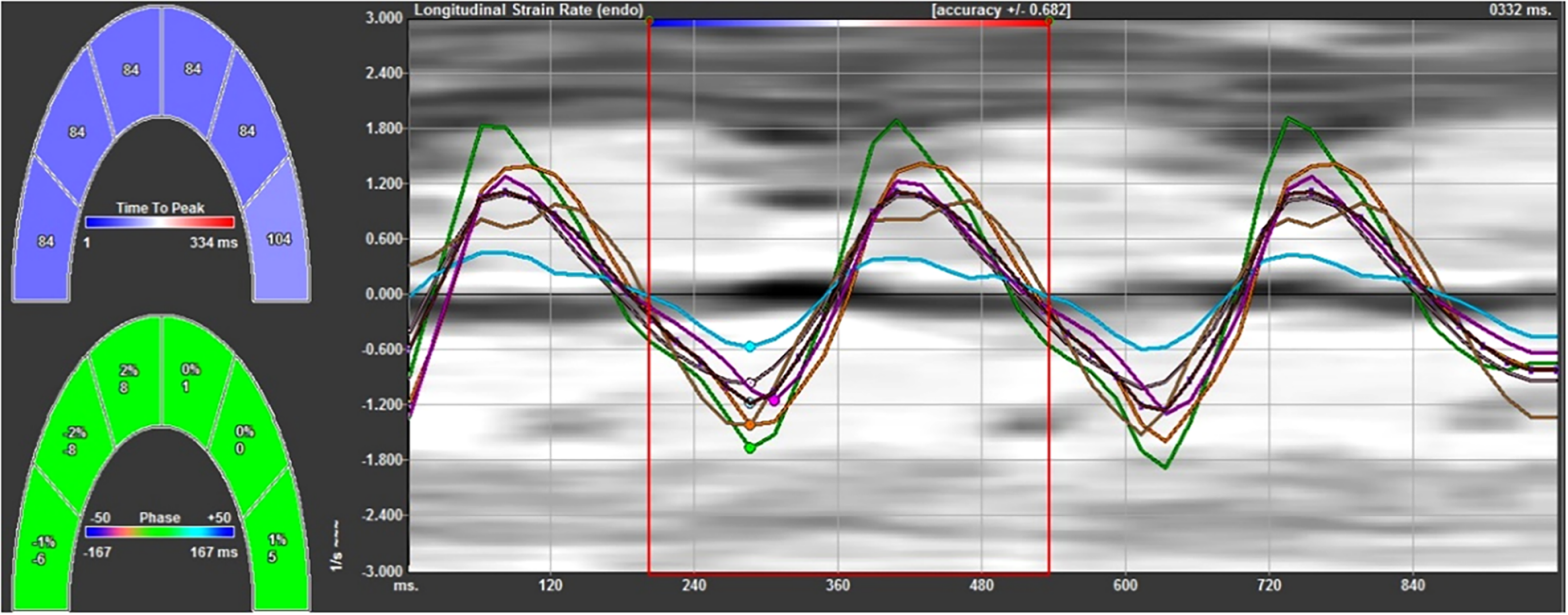
The strain rate curves of the left ventricular wall and left ventricular interventricular septum at each segment. The negative systolic strain rate indicates the longitudinal shortening of the myocardium, and the positive diastolic strain rate indicates the longitudinal elongation of the myocardium.

A routine obstetric ultrasound examination was used to determine the fetus number, fetal position, biparietal diameter, head circumference, abdominal circumference, femur, humerus and other fetal biological indexes to evaluate fetal growth and development, to exclude structural malformations and to estimate fetal weight (Supplementary Figure S1). A four-chamber view of the fetal cardiac apex was obtained, and it was ensured that the endocardium image was clear and legible. The fetal left and right ventricular walls were divided into 12 segments (the basal segment, the middle segment, and the apical segment of the left and right ventricles and their corresponding interventricular septum sides). The images were imported into the VVI analysis software for offline analysis to automatically obtain the data about the global and segmental myocardial velocity and the strain and strain rate (Figure 1). The static single-frame images were selected, of which the endocardium interface image was clearly displayed. Subsequently, the ventricular boundary was traced manually, with the starting and ending points located at the root of the tricuspid and mitral valve ring, respectively. Therefore, it was critical for the VVI to define the position of the valve ring root and the endocardial border. All measurements were calculated using the mean values of three cardiac cycles.

### 2.4. Statistical methods

SPSS 22.0 statistical software was used to test the normality of the measurement data; it was consistent with a normal distribution. Measurement data were expressed as the mean ± standard deviation. The *t* test was used for the comparison among the groups of different gestational ages. A paired t test was used for the comparison of the parameter values of different segments. An independent *t* test was used for the comparison of the parameter values between the left and right ventricles. An analysis of the correlation between the parameters and the gestational age and the fitting of a linear equation were also performed. A reliability analysis was performed for the inter-observer and intra-observer measurements. Statistical significance was indicated as *P* < 0.05.

## 3. Results

A total of 127 fetal images were analyzed offline, of which 120 images were successfully analyzed, with a success rate of 94.5%. The fetuses were divided into five groups according to gestational age: Group 1: 16–19^+6^ weeks (*n* = 14); Group 2: 20–23^+6^ weeks (*n* = 36); Group 3: 24–27^+6^ weeks (*n* = 37); Group 4: 28–31^+6^ weeks (*n* = 20); and Group 5: 32–35^+6^ weeks (*n* = 13).

### 3.1. Analysis of the VVI parameters of the left ventricular longitudinal myocardium in normal fetuses

#### 3.1.1. Comparison of the myocardial parameters in the left ventricular longitudinal segment

The six segments of the left ventricular myocardium included the basal segment, the middle segment and the apex segment of the left ventricular lateral wall and the basal segment, the middle segment, and the apex segment of the left ventricular interventricular septum. The velocity, strain, and strain rate of six segments of the left ventricular longitudinal systolic and diastolic myocardium are shown in Table 1.

**Figure 2:**
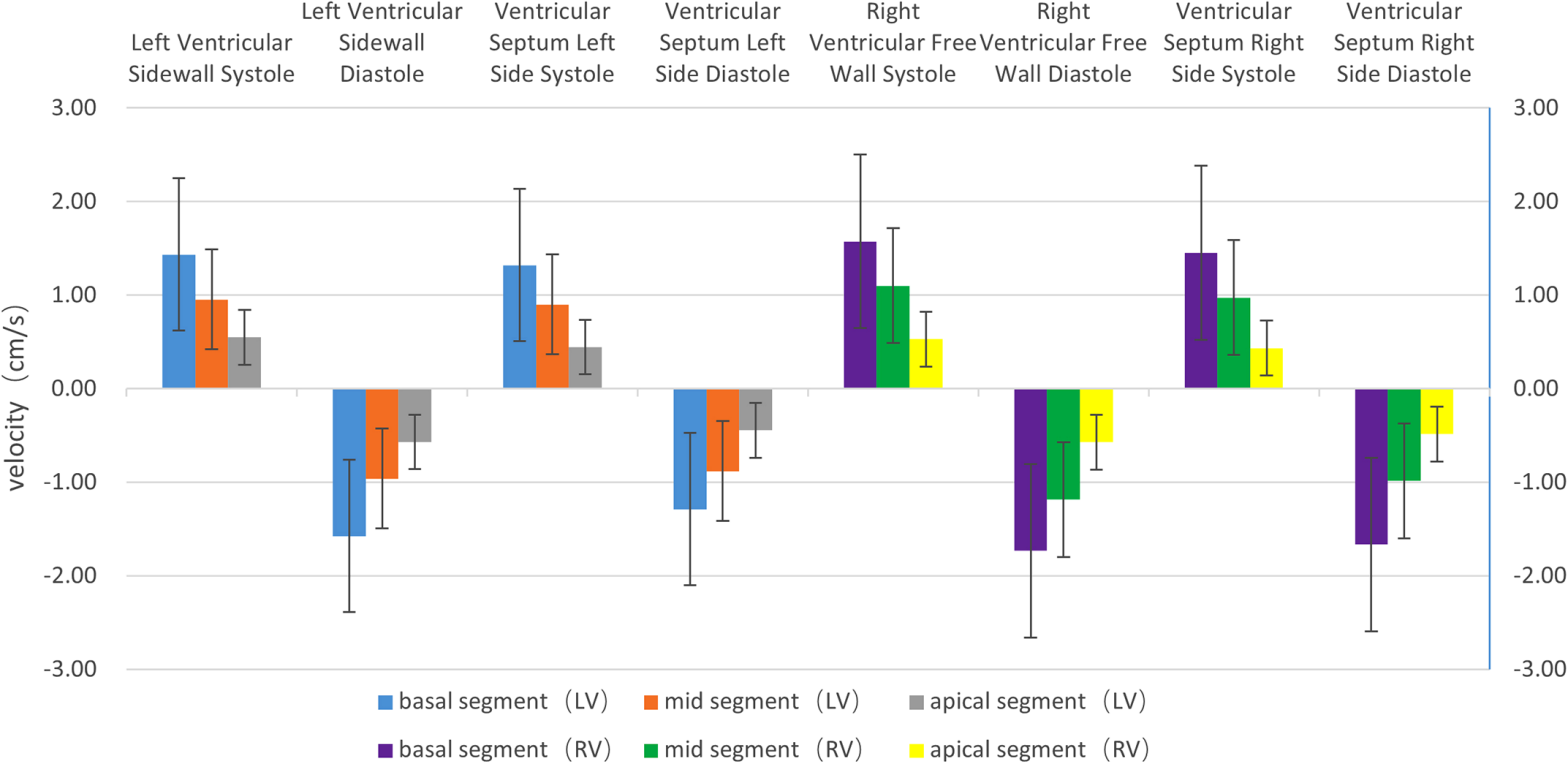
The histogram of the systolic and diastolic velocities of the left and right ventricular longitudinal walls at each segment. The velocities of the left and right ventricles, regardless of lateral wall (free wall) or interventricular septum, show a decreasing trend from the basal segment to the apex segment, in the following order: basal segment > middle segment > apex segment. The differences are statistically significant (*P* < 0.05). The systolic velocity is a positive value, and the diastolic velocity is a negative value.

**Figure 3:**
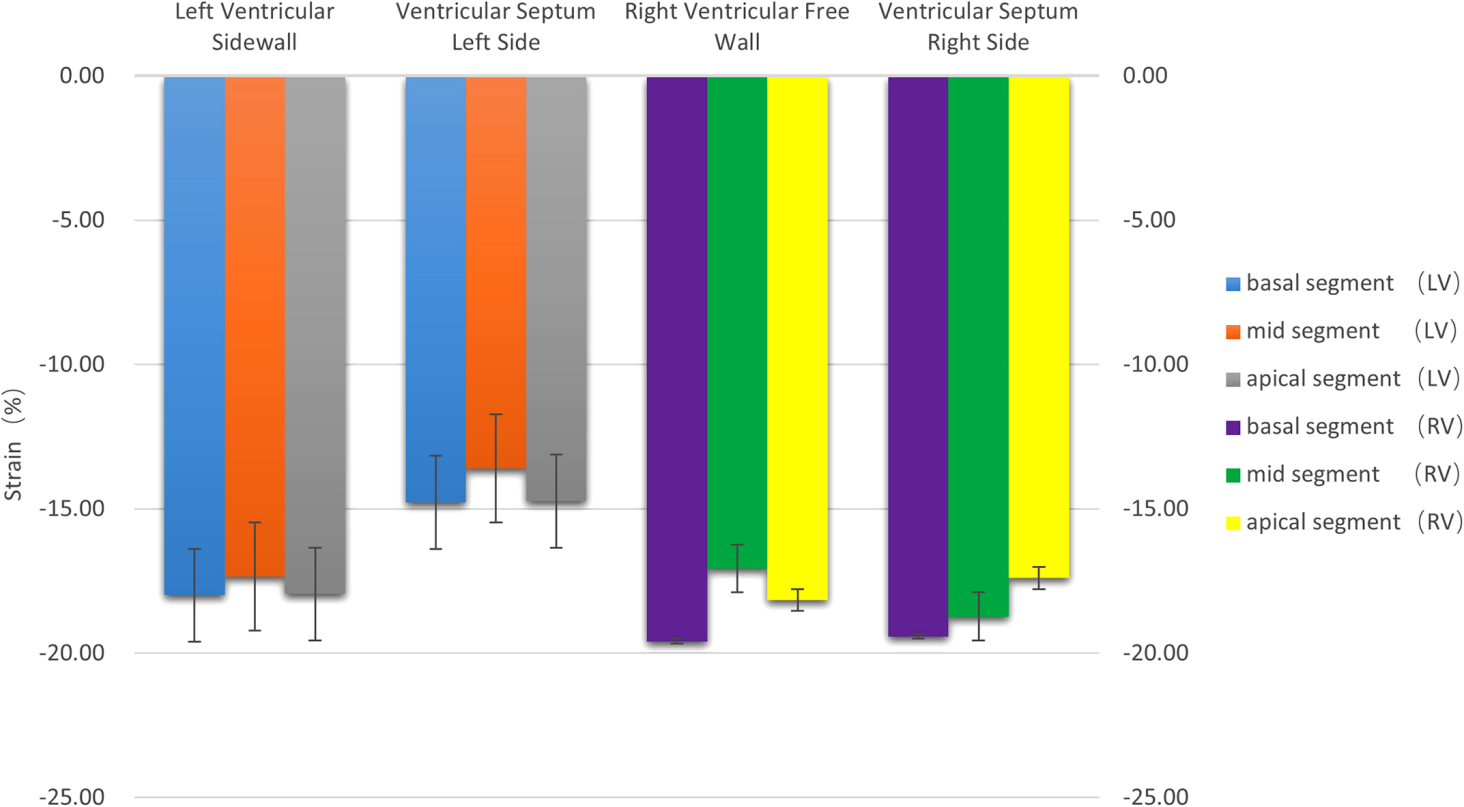
The histogram of the strain of the left and right ventricular walls at each segment. There were no statistically significant differences in the strain between the left and right ventricles (*P* > 0.05). The strain values were negative.

**Figure 4:**
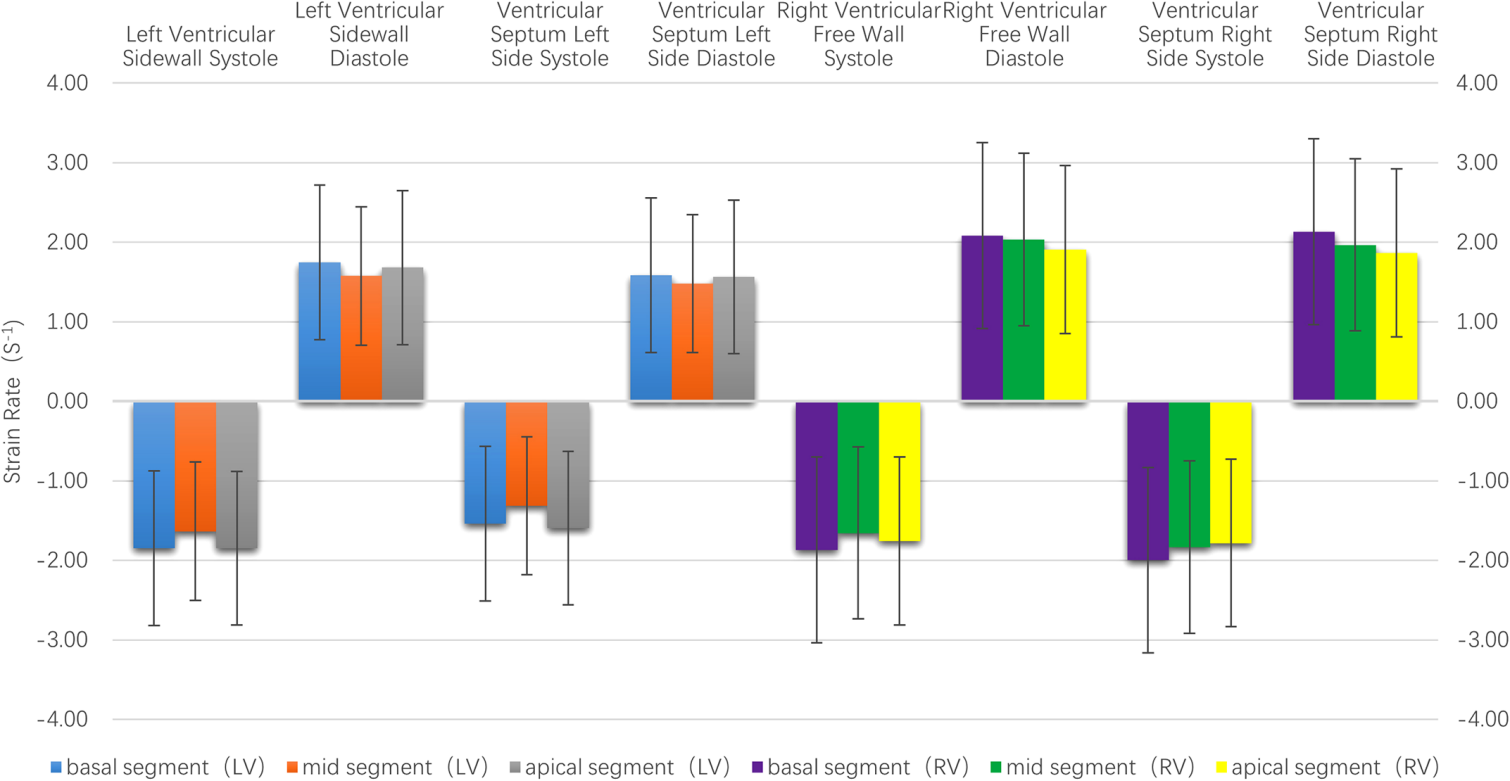
The histogram of the strain rate of the left and right ventricular wall at each segment. There were no statistically significant differences in the systolic and diastolic strain rate between the left and right ventricles at each segment. The strain rate of the right ventricle was higher than that of the left ventricle.

**(1) Velocity:** The motion velocities of the systolic and diastolic longitudinal myocardium, the lateral wall of left ventricle and the left ventricular interventricular septum, and the segmental myocardium all showed a decreasing trend from the basal to the middle to the apex segment, and the differences were statistically significant (*P* < 0.05), as is shown in Table 1 and Figure 2.
**(2) Strain:** There were no statistically significant differences in the left ventricular peak strain between the adjacent segments (*P* > 0.05), as is shown in Table 1 and Figure 3.
**(3) Strain rate:** There were no statistically significant differences in the left ventricular systolic and diastolic peak strain rate between the adjacent segments (*P* > 0.05), as is shown in Table 1 and Figure 4.

**Table 1:** Systolic and diastolic global velocity, strain and strain rate of left and right ventricular myocardium at six segments.

	Basal segment		Middle segment		Apex segment		
	Lateral wall (free wall)	Interventricular septum	Lateral wall (free wall)	Interventricular septum	Lateral wall (free wall)	Interventricular septum	Global value
**Left ventricle**
Vs	1.43 ± 0.54*	1.32 ± 0.68*	0.95 ± 0.57*	0.90 ± 0.51*	0.55 ± 0.26	0.44 ± 0.30	0.93 ± 0.61#
Vd	−1.57 ± 0.49*	−1.29 ± 0.71*	−0.96 ± 0.49*	−0.88 ± 0.61*	−0.57 ± 0.27	−0.45 ± 0.33	−0.95 ± 0.65#
S	−18.00 ± 12.55	−14.77 ± 9.79	−17.34 ± 9.37	−13.59 ± 9.79	−17.95 ± 12.50	−14.73 ± 9.99	−16.06 ± 10.85#
SRs	−1.84 ± 1.25	−1.54 ± 0.90	−1.63 ± 1.28	−1.49 ± 0.89	−1.85 ± 1.25	−1.59 ± 1.06	−1.66 ± 1.14#
SRd	1.75 ± 1.06	1.59 ± 0.81	1.58 ± 0.95	1.48 ± 1.01	1.68 ± 0.85	1.56 ± 0.88	1.61 ± 0.93#
**Right ventricle**
Vs	1.57 ± 0.66*	1.45 ± 0.64*	1.10 ± 0.48*	0.97 ± 0.60*	0.53 ± 0.30	0.43 ± 0.21	1.01 ± 0.66
Vd	−1.73 ± 0.79*	−1.67 ± 0.71*	−1.19 ± 0.65*	−0.98 ± 0.52*	−0.57 ± 0.45	−0.49 ± 0.27	−1.10 ± 0.76
S	−19.59 ± 13.29	−19.42 ± 12.79	−17.07 ± 11.99	−18.73 ± 13.81	−18.16 ± 11.02	−17.39 ± 11.07	−18.39 ± 12.37
SRs	−1.87 ± 1.19	−2.00 ± 1.73	−1.66 ± 1.36	−1.83 ± 1.46	−1.76 ± 1.15	−1.78 ± 1.21	−1.82 ± 1.36
SRd	2.08 ± 1.19	2.13 ± 1.73	2.03 ± 1.20	1.97 ± 1.46	1.91 ± 1.13	1.87 ± 1.13	2.00 ± 1.33

Vs, peak systolic velocity (cm/s); Vd, peak diastolic velocity (cm/s); S, strain (%); SRs, systolic strain rate (S^−1^); SRd, diastolic strain rate (S^−1^).

Comparison of value of this segment with that of the next segment.

* Indicted *P* < 0.05.

^#^ Represents the overall comparison between left ventricle and right ventricle, *p* < 0.05.

#### 3.1.2. Analysis on the correlation between the left ventricular longitudinal global myocardial parameters and the gestational age

**(1) Velocity:** The left ventricular systolic and diastolic global peak velocity increased with as gestational age increased.

**Figure 5:**
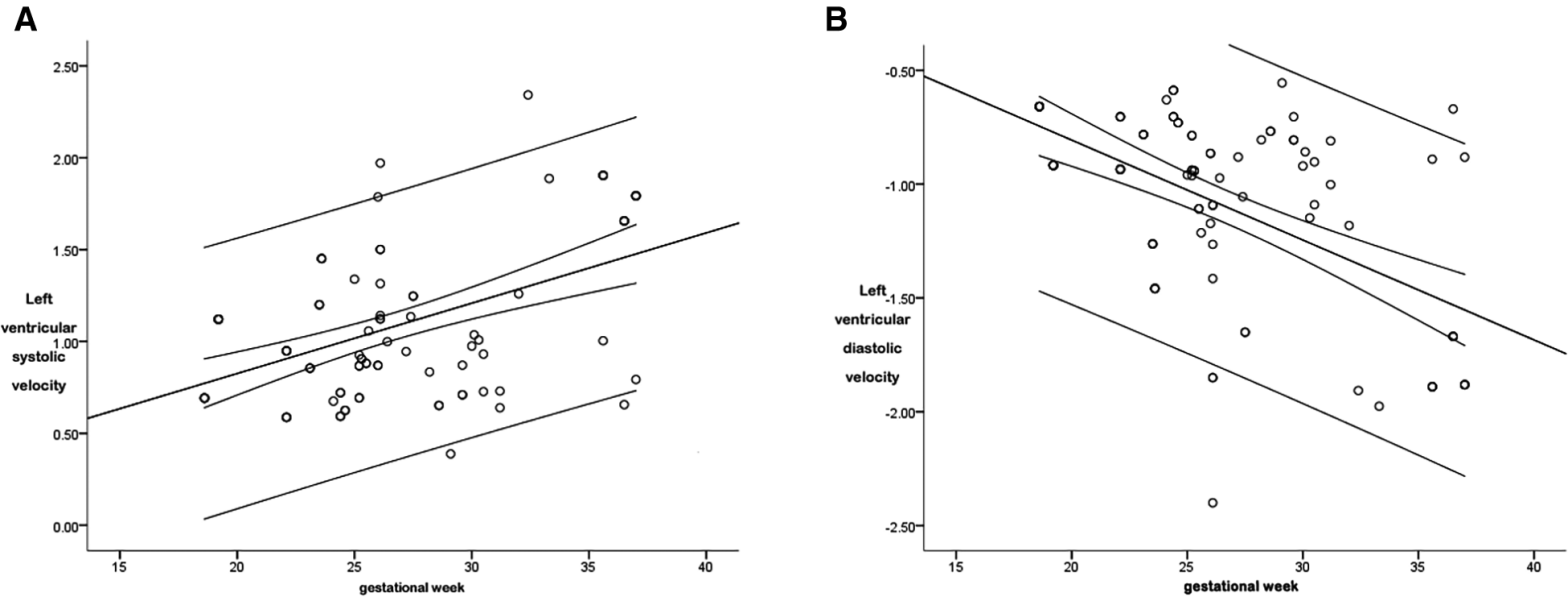
The scatter diagram of the relationship between the global left ventricular systolic (**A**) and diastolic (**B**) peak velocity and gestational age. As is shown in Figure 5A, there was a correlation between the left ventricular global systolic peak velocity and the gestational age (*P* < 0.05), with a correlation coefficient *r* = 0.497, and the regression equation was calculated as follows: global left ventricular systolic velocity = 0.061 + (0.038 × gestational age). As is shown in Figure 5B, there was a correlation between the left ventricular global diastolic peak velocity and the gestational age (*P* < 0.05), with a correlation coefficient *r* = 0.557, and the regression equation was calculated as follows: left ventricular global diastolic velocity = 0.072 + (−0.044 × gestational age).

The correlation coefficient (*r*), between the systolic global peak velocity and the gestational age, was 0.497, and the regression equation was calculated as follows: left ventricular global systolic velocity = 0.061 + (0.038 × gestational age), as is shown in Figure 5A. The regression equation is shown in Table 2.

**Table 2:** Regression equation of relationship between peak global velocities of left and right ventricular myocardium and gestational age in normal fetuses .

Variable	Velocity vs. gestational age	*r*
**Left ventricle**
Vs	Vs = 0.061 + (0.038 × ges)	0.497
Vd	Vd = 0.072 + (−0.044 × ges)	0.557
**Right ventricle**
Vs	Vs = 0.579 + (0.021 × ges)	0.369
Vd	Vd = −0.644 + (−0.022 × ges)	0.353

Vs, peak systolic velocity (cm/s); Vd, peak diastolic velocity (cm/s); *r*, correlation coefficient; ges, gestational age.

The r between the global diastolic peak velocity and the gestational age was 0.557, and the regression equation was calculated as follows: left ventricular global diastolic velocity = 0.072 + (−0.044 × gestational age), as is shown in Figure 5B. The regression equation is shown in Table 2.

**(2) Strain:** There were no statistically significant differences in the correlation between the strain and the gestational age (*P* > 0.05).
**(3) Strain rate:** There were no statistically significant differences in the correlation between the systolic and diastolic global peak strain rate and the gestational age (*P* > 0.05).

### 3.2. Analysis of the VVI parameters of the right ventricular longitudinal myocardium in normal fetuses

#### 3.2.1. Comparison of the myocardial parameters of the right ventricular longitudinal segment

The six segments of the right ventricular myocardium included the basal segment, middle segment and the apex segment of the right ventricular lateral wall and the basal segment, the middle segment and the apex segment of the right ventricular interventricular septum. The velocity, strain, and strain rate of the six segments of the right ventricular longitudinal systolic and diastolic myocardium are shown in Table 1.

**(1) Velocity:** The motion velocities of the systolic and diastolic longitudinal myocardium, the right ventricular lateral wall and the right ventricular lateral interventricular septum, and the segmental myocardium all showed a decreasing trend from the basal to the middle to the apex segment, and the differences were statistically significant (*P* < 0.05), as is shown in Table 1 and Figure 2.
**(2) Strain:** There were no statistically significant differences in the right ventricular peak strain between the adjacent segments (*P* > 0.05), as is shown in Table 1 and Figure 3.
**(3) Strain rate:** There were no statistically significant differences in the right ventricular systolic and diastolic peak strain rate between the adjacent segments (*P* > 0.05), as is shown in Table 1 and Figure 4.

#### 3.2.2. Analysis on the correlation between the right ventricular longitudinal global myocardial parameters and the gestational age

**(1) Velocity:** The right ventricular systolic and diastolic global peak velocity increased as the gestational age increased.

**Figure 6:**
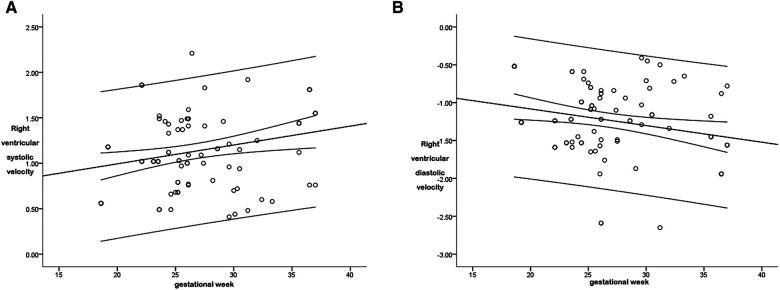
The scatter diagram of the relationship between the right ventricular global systolic (**A**) and diastolic (**B**) peak velocity and gestational age. As is shown in Figure 6A, there was a correlation between the right ventricular global systolic peak velocity and the gestational age (*P* < 0.05), with a correlation coefficient *r* = 0.369, and the regression equation was calculated as follows: right ventricular global systolic velocity = 0.579 + (0.021 × gestational age). As is shown in Figure 6B, there was a correlation between the right ventricular global diastolic peak velocity and the gestational age (*P* < 0.05), with a correlation coefficient *r* = 0.353, and the regression equation was calculated as follows: right ventricular global diastolic velocity = −0.644 + (−0.022 × gestational age).

The r between the right ventricular systolic global peak velocity and the gestational age was 0.369, and the regression equation was calculated as follows: right ventricular systolic global velocity = 0.579 + (0.021 × gestational age), as is shown in Figure 6A. The regression equation is shown in Table 2.

The *r* between the right ventricular diastolic global peak velocity and the gestational age was 0.353, and the regression equation was calculated as follows: right ventricular global diastolic velocity = −0.644 + (−0.022 × gestational age), as is shown in Figure 6B. The regression equation is shown in Table 2.

**(2) Strain:** There were no statistically significant differences in the correlation between the strain and the gestational age (*P* > 0.05).
**(3) Strain rate:** There were no statistically significant differences in the correlation between the systolic and diastolic global peak strain rate and the gestational age (*P* > 0.05).

## 4. Discussion

In this study, ultrasonic VVI was used for manual tracing on the basis of the 2D ultrasound image of a standard four-chamber view of the fetal heart to obtain the parameter values of peak velocity, peak strain, and strain rate of the left and right ventricular systolic and diastolic myocardial longitudinal segment and the global myocardium. A subsequent analysis and summary of the above parameters were performed to investigate the variation and their correlation with gestational age.

The novel echocardiographic technique of VVI is based on a 2D gray scale, which has been applied to quantitatively evaluate the deformation function of the segmental and global myocardium, including several parameters (i.e., velocity, strain, and strain rate) (5). Note that VVI can track the geometry of tissue motion changes through ultrasonic spot tracking technology and achieve the direct analysis and acquisition of the motion of the tissues by integrating frames and subtle frame changes (5). The velocity vector of a partial chamber wall motion can be directly superimposed on the 2D image in offline analysis (5).

The following parameters are involved in VVI (6): (1) Velocity (V): It refers to the displacement in unit time, namely, the speed of the position change of tissues, in cm/s; (2) Strain (S): It refers to myocardial deformation, namely, the change fraction of the myocardial segment length. The strain parameter is unitless, and it is usually expressed as a percentage. In addition, strain may be positive or negative, reflecting elongation and shortening, respectively. For example, a 10-cm rope stretched to 12 cm has a positive strain of 20%; (3) Strain rate (SR): It refers to the change rate of strain, in 1/s or s^−1^. An important advantage of both strain and strain rate is that both of them are independent of translational motion and can reflect segmental function. In general, strain describes the magnitude and direction of local myocardial shortening or lengthening. More accurately, “global strain” is “global longitudinal strain” or “global circumferential strain” and generally refers to the mean longitudinal or circumferential strain of the global myocardium. It can be approximated to the mean strain of each ventricular wall segment.

According to this study's results, the systolic segmental peak velocities of the fetal ventricles and interventricular septum showed a decreasing trend from the basal to the middle to the apex segment. The diastolic peak velocity was highest in the basal segment, followed by the middle segment, and then the apex segment. The global right ventricular peak velocity was higher than the global left ventricular peak velocity. The differences in all the above parameters were statistically significant. This was because the motion of the cardiac basal segment was more intense than the middle and apex segments. The change in the segmental myocardium velocity was associated with muscle fiber strike and cardiac swing. The high displacement amplitude and myocardial velocity in the basal segment of the free wall might be associated with the dominance of longitudinal fibers. In terms of the fiber composition in a normal heart, the right ventricular free wall is dominated by longitudinal/oblique fibers, while the left ventricular lateral wall is dominated by annular mean fibers. However, the interventricular septum consists of both fiber types. Moreover, the fiber shortening degree in the right ventricular sinus is greater than in the infundibulum; therefore, the role of the sinus and the infundibulum are not balanced for the right ventricular global function. This is also the reason for the large deviation of the right ventricular free wall. Kim et al. (7) conducted a comparative study on fetal left and right ventricular functions and found that the systolic and diastolic peak velocities of the ventricular and interventricular septal segmental myocardium showed a decreasing trend from the basal to the apex segment using the VVI technique. Domestic scholars (8) have analyzed and studied the peak velocity of fetal left ventricular segmental myocardium using VVI and reached the same conclusions. The results of this study are consistent with those found in these studies.

According to the results of this study, there were no statistically significant differences in the strain and strain rate of the left and right ventricular longitudinal myocardial segments between the adjacent ones, indicating that the deformability of each fetal ventricular segment did not change significantly. This result was consistent with that of previous studies (9). In addition, the variation trend is similar to that in adults, namely, the peak strain and peak strain rate did not change significantly in the basal segment, the intermediate segment and the apex segment.

Johnson et al. (10) measured the fetal intraventricular pressure and found that the right ventricular systolic pressure of normal fetuses increased with the gestational age. Since the development of cardiac function is almost mature in the middle trimester (11), the gradually increasing systolic pressure can only be overcome by increasing the number of cardiomyocytes and the contractile capacity of the unit myocardium when the right ventricular deformation ability remains unchanged (12). On the other hand, the increase of myocardial contractility would inevitably lead to the increase of diastolic ventricular suction. In the case of a relatively constant cardiac cycle, the instantaneous systolic and diastolic ventricular displacement would increase, resulting in an increase in the peak velocity with gestational age. The results of this study are consistent with those of previous studies.

In recent years, studies have been conducted to explore the correlation between bilateral ventricular longitudinal global strain, strain rate, and the gestational age in normal fetuses. However, the existing data on these normal values are inconsistent, and most studies are limited to a single ventricle or a small sample size (usually <100). This study not only investigated the segmental and global strain and strain rate of the left and right ventricles in a large sample size but also involved the middle and late trimesters. The changes in global strain and strain rate with gestational age found in this study are consistent with those found in some previous studies (7, 13), namely, that they will not change with the gestational age. The fetal cardiac function is mature and constant in the middle and late trimesters. According to the formula for calculating strain rate, SR = S/Δt = ΔL/L_0_/Δt = ΔL/Δt/L_0_ = ΔV/L_0 _= (V1−V2)/L_0_ (Δt, time change; ΔV, velocity change; L_0_, initial length), strain is stable in the middle and late trimesters, and the SR will not change with the increase in gestational age if the fetal heart rate fluctuates within the normal range. Therefore, strain is time-independent, and it reliably reflects the myocardial deformation. Other studies have shown that the segmental strain of the interventricular septum and ventricular free wall is linearly correlated with the gestational age (14). In addition, the strain of the left ventricle is higher than that of the right ventricle throughout the pregnancy, and this is more obvious during the late trimester. However, the SRs in both ventricles are similar and gradually decreased with the gestational age (15). The differences may be due to the use of different ultrasound systems and software packages in myocardial deformation analysis, or a small fetal sample size, or fetuses mostly in the middle or late trimester.

An accurate assessment of fetal cardiac function will contribute to the confirmation and improvement of the understanding of the course of fetal disease and its impact on the fetal heart. Echocardiographic measurements of myocardial strain have been proven accurate in evaluating mild myocardial dysfunction and regional wall motion abnormality (16–20).

This study was limited by the low frame rate of the VVI; this may have led to information loss. Therefore, increasing the frame rate without affecting the spatial resolution will enable a more accurate analysis of myocardial function with higher resolution. In addition, VVI technology could achieve speckle tracking; however, the requirement for image quality is high, and low-quality images constitute one of the main reasons for software analysis failure. The acoustic shadow formed by calcified bones during examination, especially in late pregnancy, would seriously affect image clarity, so the 2D images selected should be as clear as possible. Nevertheless, compared with a previous study (21), our study has shown that the global strain and peak strain rate did not change as gestational age increased, indicating that the myocardial deformability of the fetus' ventricles was constant in the middle and late trimesters.

## 5. Conclusions

In normal gestational fetuses, the V of the left and right ventricles show a decreasing trend from the basal segment to the apical segment. The longitudinal global myocardial systolic velocity and diastolic velocity of the left and right ventricles increase along with the fetal gestational age. In addition, the longitudinal global strain, systolic strain rate, and diastolic strain rate remain stable in the middle and late trimesters and do not change with the increase of gestational age in both the left and right ventricles, indicating that the myocardial deformability of the left and right ventricles is constant in the middle and late trimesters.

## Data Availability

The original contributions presented in the study are included in the article/**Supplementary Material**, further inquiries can be directed to the corresponding author.
