# Supplementary figures and images for: To investigate the correlation between normal fetal biventricular myocardial function and gestational age using velocity vector imaging
Source: Front Cardiovasc Med. 2023 Mar 23;10:920965. doi: 10.3389/fcvm.2023.920965 (PMC10076844; doi:10.3389/fcvm.2023.920965)

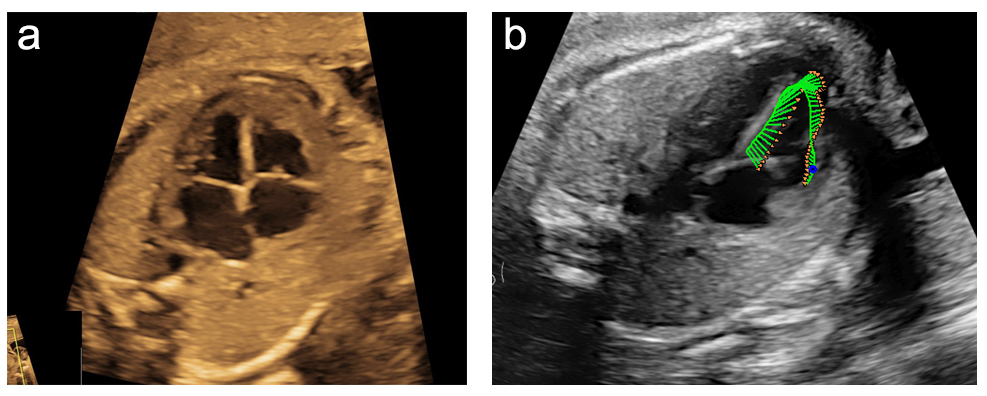

Supplement: Supplementary file 1 [file Image1.jpeg]
